# A Practical Framework to Design Immunization Studies Based on the Beta Distribution

**DOI:** 10.1002/sim.70293

**Published:** 2025-10-07

**Authors:** Stefan Embacher, Andrea Berghold, Kirsten Maertens, Sereina A. Herzog

**Affiliations:** ^1^ Institute for Medical Informatics, Statistics and Documentation Medical University of Graz Graz Austria; ^2^ Centre for the Evaluation of Vaccination, Vaccine and Infectious Diseases Institute University of Antwerp Antwerp Belgium

**Keywords:** antibody kinetics, immunization study, optimal sampling schedule, study design

## Abstract

An optimally designed experiment reaches results quicker, at a lower cost, or with fewer observations and is therefore crucial in maximizing resource efficiency in research. In immunization studies, the primary goal is often to characterize antibody kinetics—the change in antibody concentration over time. However, nonlinear models for antibody kinetics present substantial challenges for study design, particularly the need to provide information on the parameters of interest. We propose a novel framework to facilitate the design of immunization studies using simple, understandable information. We assume that the mean antibody concentration follows the structural form of the beta density until reaching a plateau. Using the time and height of the maximum and the time and height of the plateau, we can uniquely determine the antibody kinetics curve. Optimal sampling schedules are determined using D‐optimality, with D‐efficiency used to compare designs. In a robustness analysis across 12 scenarios, we analyzed the framework's sensitivity to misspecification in the initial information. When misspecifying one parameter at a time, the median D‐efficiencies exceeded 0.95 and the first quartiles were greater than or equal to 0.9 for all parameters, highlighting the robustness of the framework. Misspecification in the height of the plateau and time of the maximum affected the D‐efficiency the most. The great advantage of the framework is that we only need intuitive information from the medical professionals to design an immunization study, in which determining the antibody kinetics is the main goal.

## 1 Introduction

In immunization studies, the primary objective is often to describe antibody kinetics, that is, changes in antibody concentrations over time [1]. Typically, one assumes that the antibody concentration over time is distributed around a continuous and often nonlinear mean concentration μ(t,θ). These μ originate from various modeling approaches, typically derived from mechanistic or phenomenological foundations, modeling the underlying biological system or observed behavior through the functional form, respectively [1]. However, such models are often complex, purely theoretical, include unmeasured parameters, present identifiability issues or are difficult to interpret [2, 3, 4]. Consequently, they present significant challenges in study design and create a gap between theory and practical application.

Furthermore, resources such as time, funding, materials, or the availability of patients are often limited in scientific research [5, 6]. The theory of optimal design, which aims to improve design efficiency, has a history of around 100 years [7]. Its central idea is to identify a study design that optimizes specific optimality criteria, enabling a more efficient study that achieves the same results as a suboptimally designed study, but in less time, at a lower cost, or with fewer observations [8, 9]. Prominent optimality criteria often map the Fisher information matrix (FIM) to a scalar [8, 9]. For example, D‐optimality maximizes the determinant of the FIM, while E‐optimality minimizes the largest eigenvalue of the inverse of the FIM. D‐optimality is arguably the most popular criterion, primarily due to its intuitive graphical interpretation and ease in explanation and a generally strong performance in comparison with other criteria [8, 9, 10]. In the case of nonlinear models, however, the FIM depends on the unknown model parameters [10, 11]. Various strategies address this issue, with locally optimal designs, that is, designs based on guesses of the unknown parameters, being probably the easiest/practical approach when designing a study [9, 12]. Optimal design for nonlinear models is applied across different fields, including pharmacokinetics, a discipline closely related to our area of interest, though distinct in the specific modeling objectives [9, 13, 14]. Particularly in the field of medicine, there is an ethical responsibility to ensure the best possible design of studies.

Despite its clear advantages, optimal design theory is still underutilized in practice [15]. In our ongoing, nearly completed systematic review, we have not (yet) identified any publications that use the functional form of the antibody kinetics to actually design studies, that is, to determine the optimal number or timing of blood sample collection [16]. We argue that this underutilization is potentially due to a lack of awareness, as well as the analytical and numerical complexity involved in combining complex antibody kinetic models with optimal design theory. Additionally, the need to provide information on the parameters of interest might be very challenging, especially for complex mathematical models.

In our work, we introduce a framework that enables healthcare professionals to design an immunization study in a convenient way, using initial information that is easily interpretable. Based on the phases of methodological research in biostatistics, as defined by Heinze et al. [17], we consider this work to be Phase I. We start by introducing the idea, based on the assumption of an initial increase followed by a decrease in antibody concentrations, in the simplest form in Section 2.1. This idea is then extended to accommodate scenarios where antibody concentrations do not start at zero in Section 2.2. In Section 2.3, we describe the process of determining optimal sampling times within our framework and discuss the implementation in Section 2.4. In Section 2.5, we describe how the robustness of our framework is evaluated with respect to potential misspecification in the provided initial information by the medical professionals, including the description of the different scenarios. In Section 3, we present the results of the robustness analysis and we end with a discussion in Section 4.

## 2 Methods

### 2.1 Idea

**FIGURE 1:**
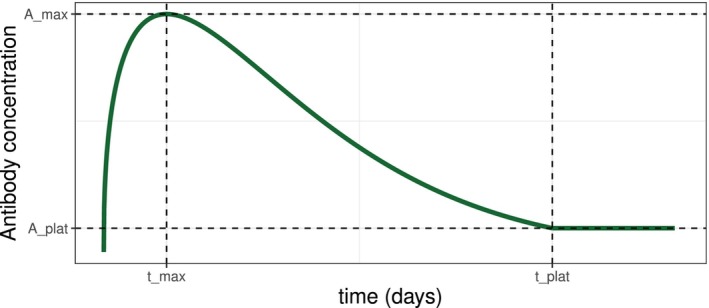
Graphical display of the basic idea of using the time of maximum $t_{\max}$, the height of the maximum $A_{\max}$, the time of the plateau $t_{\text{plat}}$, and the height of the plateau $A_{\text{plat}}$ to describe the change in antibody concentration over time.

Our idea is based on the main assumption, that the antibody concentration A(t) over time t is normally distributed around a mean antibody concentration μ(t,θ), which follows the structural form of a beta density until it reaches a plateau. This implies that antibody levels either stabilize at a specific threshold or decline to undetectable levels. We restrict the shape parameters of the beta distribution to be α>1 and β>1 in order to utilize the functional form of the beta distribution and assure an initial increase, followed by a decrease in antibody concentrations, as displayed in Figure 1. We begin with patients who have undetectable antibody concentrations at time t=0, that is, A(0)=0. For the beta distribution and the respective choice of parameters, it is then known that the mode $x_{\max}$ is given by [18]:

$$x_{\max}=\frac{\alpha -1}{\alpha +\beta -2}$$

and that the probability density function f(x,α,β) is given by

$$f\left(x,\alpha ,\beta \right)=\frac{1}{B\left(\alpha ,\beta \right)}x^{\alpha -1}{\left(1-x\right)}^{\beta -1}$$

where B(α,β) denotes the beta function. The mean antibody concentration μ(t,θ) can be uniquely described and determined using simple and easily understandable input from the medical professionals. Namely, the time of the maximum antibody concentration $t_{\max}$, the (expected) value of the maximum antibody concentration $A_{\max}=E\left[A\left(t_{\max}\right)\right]=\mu \left(t_{\max},\theta \right)$, the time at which the plateau is reached $t_{\text{plat}}$, and the corresponding (expected) value of the plateau $A_{\text{plat}}=E\left[A\left(t_{\text{plat}}\right)\right]=\mu \left(t_{\text{plat}},\theta \right)$. Figure 1 gives a graphical display of the underlying idea of using these initial information. Since the beta distribution is defined over the interval [0,1], an additional scaling parameter $t_{\text{scale}}$ is introduced to map time onto a useful scale, such as days, thereby ensuring that the framework remains applicable and interpretable in real world scenarios. Through this scaling parameter we assure via $x_{\max}=\frac{t_{\max}}{t_{\text{scale}}}$ and $x_{\text{plat}}=\frac{t_{\text{plat}}}{t_{\text{scale}}}$ that $x_{\max}\in \left[0,1\right]$ and $x_{\text{plat}}\in \left[0,1\right]$, with the restriction that $t_{\text{scale}}\geq t_{\text{plat}}>t_{\max}$.

We assume that the antibody kinetics follow the given structure

(1) $$E\left[A\left(t\right)\right]=\mu \left(t,\theta \right)=\left(\begin{array}{ll} f\left(\frac{t}{t_{\text{scale}}},\alpha ,\beta \right), & \text{for}\;0<t<t_{\text{plat}}\\ A_{\text{plat}}, & \text{for}\;t\geq t_{\text{plat}} \end{array}\right.$$

By the provided information, we can uniquely determine μ(t,θ) and result in the following system of equations:

(2) $$\left(\begin{array}{cc} 0 & =\frac{\alpha -1}{\alpha +\beta -2}-\frac{t_{\max}}{t_{\text{scale}}}\\ f\left(t_{\max},\alpha ,\beta ,t_{\text{scale}}\right)=A_{\max} & =\frac{1}{B\left(\alpha ,\beta \right)}{\left(\frac{t_{\max}}{t_{\text{scale}}}\right)}^{\alpha -1}{\left(1-\frac{t_{\max}}{t_{\text{scale}}}\right)}^{\beta -1}\\ f\left(t_{\text{plat}},\alpha ,\beta ,t_{\text{scale}}\right)=A_{\text{plat}} & =\frac{1}{B\left(\alpha ,\beta \right)}{\left(\frac{t_{\text{plat}}}{t_{\text{scale}}}\right)}^{\alpha -1}{\left(1-\frac{t_{\text{plat}}}{t_{\text{scale}}}\right)}^{\beta -1} \end{array}\right.$$

which we need to solve for α,β, and $t_{\text{scale}}$.

### 2.2 Extension

One of the underlying assumptions in (2) is A(0)=0. However, this assumption is often violated, for example, in patients with prior exposure to vaccination or antigens or in newborns who have acquired maternal antibodies through transplacental transfer [19, 20, 21]. To address this, we extend our framework to incorporate the possibility of modeling A(0)>0 in the antibody kinetics, by introducing a shift parameter c. Despite the shift, we still require the model to show an initial increase in antibody concentrations. By the restriction of the parameters α>1 and β>1, which directly implies that f(x,α,β)=0 for x=0, we need to fulfill c>0, while A(0)=0 implies c=0. Analogously to $x_{\max}$ and $x_{\text{plat}}$, we introduce $x_{c}=\frac{c}{t_{\text{scale}}}$. Therefore, with the additional information of where we expect to start $A_{0}$, we result in the following system of equations:

(3) $$\left(\begin{array}{l} \begin{array}{cc} 0 & =\frac{\alpha -1}{\alpha +\beta -2}-\frac{t_{\max}+c}{t_{\text{scale}}}\\ f\left(\alpha ,\beta ,t_{\text{scale}},c\right)=A_{0} & =\frac{1}{B\left(\alpha ,\beta \right)}{\left(\frac{c}{t_{\text{scale}}}\right)}^{\alpha -1}{\left(1-\frac{c}{t_{\text{scale}}}\right)}^{\beta -1}\\ \;f\left(t_{\max},\alpha ,\beta ,t_{\text{scale}},c\right)=A_{\max} & =\frac{1}{B\left(\alpha ,\beta \right)}{\left(\frac{t_{\max}+c}{t_{\text{scale}}}\right)}^{\alpha -1}{\left(1-\frac{t_{\max}+c}{t_{\text{scale}}}\right)}^{\beta -1}\\ f\left(t_{\text{plat}},\alpha ,\beta ,t_{\text{scale}},c\right)=A_{\text{plat}} & =\frac{1}{B\left(\alpha ,\beta \right)}{\left(\frac{t_{\text{plat}}+c}{t_{\text{scale}}}\right)}^{\alpha -1}{\left(1-\frac{t_{\text{plat}}+c}{t_{\text{scale}}}\right)}^{\beta -1} \end{array} \end{array}\right.$$

which we need to solve for α,β, $t_{\text{scale}}$, and c.

### 2.3 Solve for Optimal Design

To find the optimal time‐points within our framework, we will use ideas from optimal design theory, where the FIM is of crucial importance [8, 9]. We assume that we have p parameters, denoted as $\theta ={\left(\theta_{1},\ldots ,\theta_{p}\right)}^{T}$, and a time‐dependent mean with n measurements $\mu \left(t,\theta \right)={\left(\mu \left(t_{1}\theta \right),\ldots ,\mu \left(t_{n}\theta \right)\right)}^{T}$. In our framework θ is either the vector ${\left(\alpha ,\beta ,t_{\text{scale}}\right)}^{T}$ or ${\left(\alpha ,\beta ,t_{\text{scale}},c\right)}^{T}$ and the mean μ(t,θ) is given as described in Equation (1). We assume that the measurements are multivariate normal $Y∼N_{n}\left(\mu \;\left(t\theta \right),\sum \left(\theta \right)\right)$, while making the simplification that the covariance matrix is constant, that is, ∑(θ)=∑. Then, we know that the (i,j) entry of the FIM is given by [9, 22]

$$\mathrm{FI}M_{\mathrm{ij}}=\frac{\partial \mu^{T}}{\partial \theta_{i}}\sum^{-1}\frac{\partial \mu }{\partial \theta_{j}}$$

The partial derivatives of the beta density, as we have adapted it, are given as follows:

$$\left(\begin{array}{l} \begin{array}{cc} \frac{\partial \mu }{\partial \alpha } & =\frac{1}{B\left(\alpha ,\beta \right)}\left[\log\left(\frac{t+c}{t_{\text{scale}}}\right){\left(1-\frac{t+c}{t_{\text{scale}}}\right)}^{\beta -1}{\left(\frac{t+c}{t_{\text{scale}}}\right)}^{\alpha -1}\right.\\ & \;\left.-\;\left(\psi \left(\alpha \right)-\psi \left(\alpha +\beta \right)\right){\left(1-\frac{t+c}{t_{\text{scale}}}\right)}^{\beta -1}{\left(\frac{t+c}{t_{\text{scale}}}\right)}^{\alpha -1}\right]\\ \frac{\partial \mu }{\partial \beta } & =\frac{1}{B\left(\alpha ,\beta \right)}\left[\log\left(1-\frac{t+c}{t_{\text{scale}}}\right){\left(1-\frac{t+c}{t_{\text{scale}}}\right)}^{\beta -1}{\left(\frac{t+c}{t_{\text{scale}}}\right)}^{\alpha -1}\right.\\ & \;\left.-\;\left(\psi \left(\beta \right)-\psi \left(\alpha +\beta \right)\right){\left(1-\frac{t+c}{t_{\text{scale}}}\right)}^{\beta -1}{\left(\frac{t+c}{t_{\text{scale}}}\right)}^{\alpha -1}\right]\\ \frac{\partial \mu }{\partial t_{\text{scale}}} & =\frac{1}{B\left(\alpha ,\beta \right)t_{\text{scale}}^{2}}\left[\left(\beta -1\right)\left(t+c\right){\left(1-\frac{t+c}{t_{\text{scale}}}\right)}^{\beta -2}{\left(\frac{t+c}{t_{\text{scale}}}\right)}^{\alpha -1}\right.\\ & \;\left.-\;\left(\alpha -1\right)\left(t+c\right){\left(1-\frac{t+c}{t_{\text{scale}}}\right)}^{\beta -1}{\left(\frac{t+c}{t_{\text{scale}}}\right)}^{\alpha -2}\right]\\ \frac{\partial \mu }{\partial c} & =\frac{1}{B\left(\alpha ,\beta \right)t_{\text{scale}}}\left[\left(\alpha -1\right){\left(1-\frac{t+c}{t_{\text{scale}}}\right)}^{\beta -1}{\left(\frac{t+c}{t_{\text{scale}}}\right)}^{\alpha -2}\right.\\ & \;\left.-\;\left(\beta -1\right){\left(1-\frac{t+c}{t_{\text{scale}}}\right)}^{\beta -2}{\left(\frac{t+c}{t_{\text{scale}}}\right)}^{\alpha -1}\right] \end{array} \end{array}\right.$$

where ψ(z) denotes the digammafunction and B(α,β) is the beta function. In the simpler case where A(0)=0 and therefore c=0, the system slightly simplifies as shown in Data S3.

To optimize the properties of a design, we need to introduce an optimality criterion [9]. We opt for D‐optimality, which maximizes the determinant of the FIM [9, 23], which graphically corresponds to minimizing the volume of the confidence ellipsoid [9]. Since the D‐optimality criterion, where we only use the determinant of the FIM, does not fulfill the convexity property, we use −ln(det(FIM)+1) instead [9]. We have added a constant of 1 in this transformation to ensure that designs with det(FIM)=0 correspond to an objective function value of zero, while also improving the numerical stability if det(FIM) is very small. Under the assumption of one true parameter vector, which we have by the fact that we are using locally optimal designs, D‐optimal designs imply that the number of samples are equal to the number of system parameters. Taking additional samples would result in replicates of the original samples [24]. This means that the FIM is a 3 × 3 matrix in the case when A(0)=0 and a 4 × 4 matrix if A(0)>0, due to the extra parameter c. To compare different designs, we will use D‐efficiency, with values ranging between 0 and 1, where higher values indicating more favorable designs [8]. In our framework, D‐efficiency can be interpreted as the relative efficiency of a design compared to the optimal design, for example, a D‐efficiency of 0.5 corresponds to a design with 50% efficiency of the corresponding optimal design.

### 2.4 Implementation

#### 2.4.1 Beta Parameters

Based on the defined systems of Equations (2) and (3), that is, whether A(0)=0 or not, we solve for three or four parameters. We solve the respective system of equations using the R‐Package “nleqslv,” Version 3.3.5 [25]. We use both, the implemented Broyden and Newton method with the default global strategy of “double dogleg,” based on the package's authors argumentation [25, 26]. We used default values for all tolerances, automatic scaling and a maximum of 1000 iterations. The solutions are only used if the algorithm converges and fulfills the restrictions on the parameters (e.g., α > 1).

#### 2.4.2 Optimal Design

To identify the optimal solution, that is, the optimal time‐points, we use the Hooke and Jeeves method, a pattern search procedure used to minimize nonlinear functions not relying on gradients [27], implemented as the function “hjn” in the R‐package “optimx” version 2023‐10.21 [28, 29]. As starting values for the optimization algorithm, we chose Day 1, the provided time of maximum $t_{\max}$ and time of the plateau $t_{\text{plat}}$. If we do not start in zero, we additionally add the fourth starting time as the middle between $t_{\max}$ and $t_{\text{plat}}$, that is, $t_{\max}+\left(t_{\text{plat}}-t_{\max}\right)/2$. One benefit of the Hooke and Jeeves method is that it allows for constraints on lower and upper bounds of the time‐points, which is useful when designing a study. This option allows clinicians to predefine time‐windows for sample collection (e.g., patients have a fixed visit after 2 weeks post‐intervention). Clearly, all time‐points must be greater equal to 0 and should be before the actual end of the study, which we assume is at the latest at the time when the plateau is reached. For numerical reasons, we set the number of function evaluations to 100 000 and increased the stepsize by a factor of 1.5 compared to default.

### 2.5 Robustness Analysis

To assess the robustness and other key properties of our proposed framework, we conducted a robustness analysis. For that purpose, we made assumptions on the variance–covariance matrix, defined various scenarios and ranges of potential misspecification. We analyzed the framework's sensitivity to misguesses in the initial information, as they typically occur at the planning stage of any (immunization) study. The robustness analysis is done in R, Version 4.4.1 [30].

#### 2.5.1 Scenarios

We defined 12 scenarios with the aim to cover a broad range of possibilities in the world of antibody kinetics following exposure. We fixed the value of $A_{\max}=10$, to be a reference for the other antibody levels, which can be interpreted as relative to the maximum. We vary whether we start in zero, at a lower level or a higher level. Further, we distinguish between faster and slower increases in antibody levels by varying the timing of the maximum as well as between lower and higher plateaus. To calculate the FIM, we make the assumption that each measurement shows the same known variability $\sigma^{2}$. We further assume that our variance–covariance matrix is that of an AR(1) model [31], where the parameter ρ is also assumed to be known. This implies that the variance–covariance matrix does not depend on the unknown parameters and is therefore constant. For the robustness analysis we fixed the standard deviation of the measurements to 0.25. Based on the data from a previous publication, we assume ρ=0.73, reflecting a strong correlation between measurements [19, 32]. In Data S3, we have shown analytically that the choices of σ and ρ do not influence the optimal sampling times. The details of the defined scenarios are summarized in Table 1. The parameters of the beta distribution and the corresponding optimal time‐points for the true scenarios are given in Table 2.

**TABLE 1:** Definition of the 12 scenarios used for the robustness analysis, where time variables are given in days and antibody concentration in arbitrary units.

Scenario Nr.	Description	*A*_0_	*t*_max_	*A*_max_	*t*_plat_	*A*_plat_
1	Start in zero, faster increase, lower plateau	0	30	10	365	1
2	Start in zero, slower increase, lower plateau	0	70	10	365	1
3	Start in zero, faster increase, higher plateau	0	30	10	365	2.5
4	Start in zero, slower increase, higher plateau	0	70	10	365	2.5
5	Start at lower level, faster increase, lower plateau	1	30	10	365	1
6	Start at lower level, slower increase, lower plateau	1	70	10	365	1
7	Start at lower level, faster increase, higher plateau	1	30	10	365	2.5
8	Start at lower level, slower increase, higher plateau	1	70	10	365	2.5
9	Start at higher level, faster increase, lower plateau	5	30	10	365	1
10	Start at higher level, slower increase, lower plateau	5	70	10	365	1
11	Start at higher level, faster increase, higher plateau	5	30	10	365	2.5
12	Start at higher level, slower increase, higher plateau	5	70	10	365	2.5

Abbreviations: $A_{0}$, provided starting value; $A_{\max}$, provided maximum antibody concentration; $A_{\text{plat}}$, provided height of the plateau; $t_{\max}$, provided time of maximum antibody concentration; $t_{\text{plat}}$, provided time of plateau.

**TABLE 2:** Parameters of the beta distribution and optimal time‐points by scenario. The scenarios differ between: Starting in zero (1–4), at a lower level (5–8), and at a higher level (9–12); showing a faster increase (1, 3, 5, 7, 9, 11) or a slower increase (2, 4, 6, 8, 10, 12); and a higher (3, 4, 7, 8, 11, 12) or lower plateau (1, 2, 5, 6, 9, 10).

Scenario Nr.	*α*	*β*	*t*_scale_	*c*	Time 1	Time 2	Time 3	Time 4
1	1.24	15.81	1919.88	0.00	0.30	29.67	183.83	—
2	1.79	23.46	2056.98	0.00	11.44	69.91	211.03	—
3	1.15	14.12	2706.91	0.00	0.03	29.54	260.40	—
4	1.49	19.80	2741.19	0.00	5.54	69.76	269.56	—
5	1.24	15.81	1919.89	0.00	0.00	0.35	30.17	184.44
6	1.82	23.72	2067.73	1.63	0.00	15.02	77.01	219.17
7	1.15	14.12	2706.91	0.00	0.00	0.07	31.63	262.93
8	1.49	19.83	2742.92	0.25	0.00	7.07	73.83	274.33
9	1.24	15.92	1926.89	0.66	0.00	3.28	44.92	202.59
10	2.23	27.84	2218.27	27.31	0.00	30.80	108.87	254.92
11	1.15	14.13	2707.91	0.10	0.00	0.91	43.30	277.31
12	1.59	21.13	2812.25	10.68	0.00	21.67	108.27	313.48

Our main goal is to assess how sensitive the framework reacts to misspecification in the initial information, such as incorrect assumptions about the timing of the maximum antibody concentration. Since the purpose of the framework is to design studies, chances are high that in practice we misspecify the initial information. We made the following assumptions about the ranges of misspecification, where we denote the correct value of the respective scenario with the additional subscript _true:

- $A_{0}\in \left[\max\left(0A_{0\_\text{true}}-2\right),A_{0\_\text{true}}+2\right]$
- $A_{\max}\in \left[A_{\max\_\text{true}}-2,A_{\max\_\text{true}}+2\right]$
- $A_{\text{plat}}\in \left[\max\left(0A_{\text{plat}\_\text{true}}-2\right),A_{\text{plat}\_\text{true}}+2\right]$
- $t_{\max}\in \left[t_{\max\_\text{true}}-14,t_{\max\_\text{true}}+14\right]$
- $t_{\text{plat}}\in \left[t_{\text{plat}\_\text{true}}-50,t_{\text{plat}\_\text{true}}+50\right]$

In Scenarios 1–4, we do not vary $A_{0}$, as we assume that at the planning stage, it is known whether the population has pre‐existing antibodies and therefore, by the defined scenarios in Table 1 and the ranges of misspecification, $A_{0}$ is either fixed to 0 (Scenarios 1–4) or varies in the interval [0,3] (Scenarios 5–8) or [3,7] (Scenarios 9–12). $A_{\max}$ varies within the interval [8,12] for all scenarios and $t_{\max}$ is either varied in the interval [16,44] (Scenarios 1, 3, 5, 7, 9, and 11) or [56,84] (Scenarios 2, 4, 6, 8, 10, and 12). Additionally, $A_{\text{plat}}$ is ranging in the interval [0,3] (Scenarios 1, 2, 5, 6, 9, and 10) or [0.5,4.5] (Scenarios 3, 4, 7, 8, 11, and 12), while for $t_{\text{plat}}$ the interval is [315,415]. To examine the influence of a single information on the optimal sampling times, we fix all values to their true value, except for one which we vary by the defined ranges on a equidistant grid with N=1001 points. To calculate the D‐efficiency of the design resulting from the misspecified assumptions, we use the parameters based on the true values and compare the misspecified optimal time points with the true optimal time‐points. To analyze the impact of parameter misspecification on deviations from the optimal time‐points, we build the difference between the misspecified optimal time‐points and the true optimal time‐points. To further analyze the simultaneous misspecification of $t_{\max}$ and $A_{\text{plat}}$, we have used the same scenarios and the same ranges of misspecification. The grid size for the single information is reduced to 101, resulting in 10 201 different numerical values per scenario.

## 3 Results

To analyze the robustness of our proposed framework, we focus on the D‐efficiency of the design under misspecification of the initial information. We vary one parameter at a time while keeping the others fixed, and we determine the D‐efficiency for each misspecified parameter for each scenario.

**FIGURE 2:**
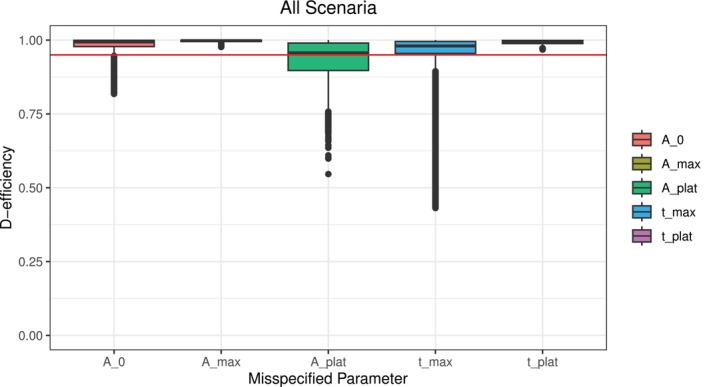
Boxplot showing the D‐efficiency of all scenarios pooled together. The red line highlights a D‐efficiency of 0.95. The misspecified parameters are the initial information used to determine the parameters of the beta distribution with misspecifications ranging in the defined ranges on a grid of size *N* = 1001 for each scenario.

Looking at the values in Table 2, we can see similarities between the different scenarios. All scenarios, that do not start with antibody levels in zero, result in a sample at time 0. In terms of parameters, we can see a pattern resulting from the combination of the speed of increase and the height of the plateau. For example, scenarios 1 and 5, both with a faster increase and a lower plateau, result in almost similar parameter values $\left(\alpha ,\beta ,t_{\text{scale}},c\right)$ and sampling times, with an extra sample at Time 0. If we compare them to Scenario 9, also faster increase with lower plateau but a higher starting value, we can see that the parameters, except for the constant c, are again very comparable. However, the resulting sampling times then differ to the other two scenarios mentioned beforehand. The same is true for the scenarios with a faster increase and a higher plateau. Scenarios 3 and 7, starting in zero and at a lower level, result in very similar parameters and sampling times while Scenario 11, starting at a higher level, results in different sampling times. In the scenarios with a slower increase and a higher plateau and starting in zero or low (Scenarios 4 and 8), we can see comparable parameter values but sampling times differ up to 5 days. However, Scenario 12, starting at a higher level, differs in terms of parameters and sampling times. The same observations can be made for Scenarios 2 and 6 (slower increase and lower plateau), with sampling times differing up to 8 days and Scenario 10 showing different results. Pooling all scenarios together, as shown in Figure 2 and Table 3, we observe that misspecifications in $A_{\max}$ influence the efficiency of a design only marginally (median 1.0, range: 0.98–1.0). The same holds for $t_{\text{plat}}$, where we observe only marginal changes in the D‐efficiency of the design when its input parameters are misspecified (median 0.99, range: 0.97–1.00). In contrast, misspecification in $A_{\text{plat}}$ (median 0.96, range: 0.55–1.0) and $t_{\max}$ (median 0.98, range: 0.43–1.0) have the strongest effect on the D‐efficiency. For the starting value $A_{0}$, we observe a median efficiency of 0.99 (range: 0.82–1.0), where especially Scenario 9, with a minimal D‐efficiency of 0.90, and Scenario 11, with a minimal D‐efficiency of 0.82, seem sensitive to misspecification (see Table 4).

**TABLE 3:** D‐efficiency for all scenarios pooled together, reported as minimum, first quartile (Q1), median, third quartile (Q3), and maximum. Additionally, the absolut and relative frequency of runs not converging are given. For $A_{0}$ we had 8008 runs, while for all others we had 12 012. For the double misspecification of $t_{\max}$ and $A_{\text{plat}}$ 122 412 runs across 12 scenarios were conducted.

Characteristics	Minimum	Q1	Median	Q3	Maximum	Not converging
*A*_0_	0.82	0.98	0.99	1	1	269 (3.4%)
*A*_max_	0.98	1	1	1	1	151 (1.3%)
*A*_plat_	0.55	0.9	0.96	0.99	1	437 (3.6%)
*t*_max_	0.43	0.95	0.98	1	1	442 (3.7%)
*t*_plat_	0.97	0.99	0.99	1	1	133 (1.1%)
*t*_max_, *A*_plat_	0.02	0.88	0.94	0.97	1	6381 (5.2%)

**TABLE 4:** The D‐efficiency for misspecification in the respective initial information and scenario. Values are reported as median (range). The scenarios differ between: Starting in zero (1–4), at a lower level (5–8), and at a higher level (9–12); showing a faster increase (1, 3, 5, 7, 9, 11) or a slower increase (2, 4, 6, 8, 10, 12); and a higher (3, 4, 7, 8, 11, 12) or lower plateau (1, 2, 5, 6, 9, 10).

	*A*_0_	*A*_max_	*A*_plat_	*t*_max_	*t*_plat_
Scenario 1	—	1.00 (0.99–1.00)	0.94 (0.60–1.00)	0.96 (0.73–1.00)	1.00 (0.98–1.00)
Scenario 2	—	1.00 (0.99–1.00)	0.95 (0.61–1.00)	0.99 (0.93–1.00)	0.99 (0.97–1.00)
Scenario 3	—	1.00 (0.98–1.00)	0.95 (0.78–1.00)	0.97 (0.91–1.00)	0.99 (0.98–1.00)
Scenario 4	—	1.00 (0.98–1.00)	0.96 (0.79–1.00)	0.99 (0.94–1.00)	0.99 (0.97–1.00)
Scenario 5	1.00 (0.98–1.00)	1.00 (0.99–1.00)	0.96 (0.66–1.00)	0.97 (0.91–1.00)	1.00 (0.98–1.00)
Scenario 6	0.99 (0.97–1.00)	1.00 (1.00–1.00)	0.96 (0.55–1.00)	0.98 (0.92–1.00)	0.99 (0.98–1.00)
Scenario 7	1.00 (0.99–1.00)	1.00 (0.99–1.00)	0.98 (0.83–1.00)	0.99 (0.94–1.00)	1.00 (0.99–1.00)
Scenario 8	1.00 (0.97–1.00)	1.00 (0.98–1.00)	0.96 (0.83–1.00)	0.99 (0.93–1.00)	0.99 (0.97–1.00)
Scenario 9	0.98 (0.90–1.00)	1.00 (0.98–1.00)	0.94 (0.69–1.00)	0.94 (0.47–1.00)	1.00 (0.98–1.00)
Scenario 10	0.99 (0.94–1.00)	1.00 (0.98–1.00)	0.97 (0.80–1.00)	0.99 (0.94–1.00)	1.00 (0.98–1.00)
Scenario 11	0.97 (0.82–1.00)	1.00 (0.98–1.00)	0.94 (0.69–1.00)	0.93 (0.43–1.00)	0.99 (0.97–1.00)
Scenario 12	0.99 (0.94–1.00)	1.00 (0.98–1.00)	0.97 (0.86–1.00)	0.99 (0.94–1.00)	0.99 (0.98–1.00)

**FIGURE 3:**
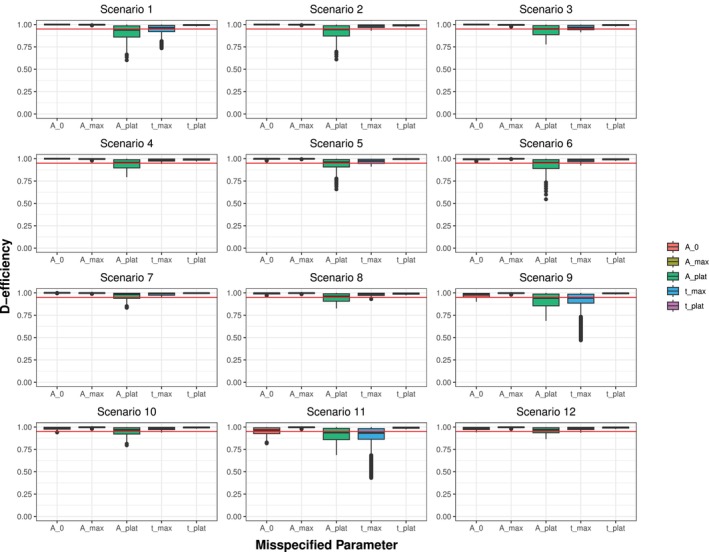
Boxplot showing the D‐efficiency of all scenarios separately. The red line highlights a D‐efficiency of 0.95. The misspecified parameters are the initial information used to determine the parameters of the beta distribution with misspecifications ranging in the defined ranges on a grid of size *N* = 1001 for each scenario. The scenarios differ between: Starting in zero (1–4), at a lower level (5–8) and at a higher level (9–12); showing a faster increase (1, 3, 5, 7, 9, 11) or a slower increase (2, 4, 6, 8, 10, 12); and a higher (3, 4, 7, 8, 11, 12) or lower plateau (1, 2, 5, 6, 9, 10).

**FIGURE 4:**
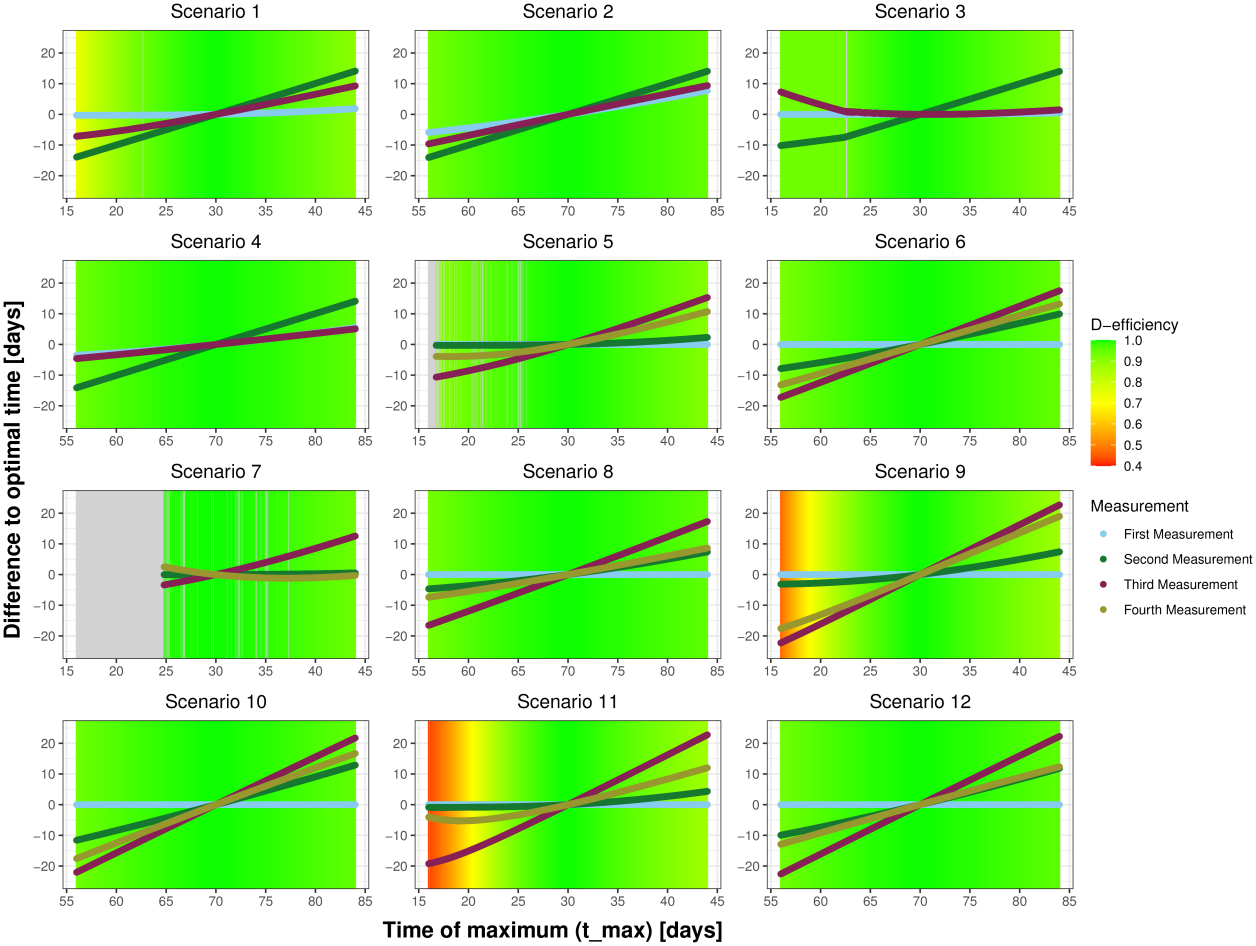
Plots illustrating the deviation of sampling times with respect to misspecification in the time of maximum $t_{\max}$. Each plot represents one scenario. The lines represent the deviation of the optimal sampling times, based on misspecified initial information, compared to the optimal sampling times resulting by the “true” initial information. The color gradient highlights the D‐efficiency of the respective design, reflecting the effect of deviation of sampling times on the D‐efficiency. In Scenarios 1–4, three time points are optimized, while in scenarios 5–12, four time points are optimized. The gray area indicates settings where no solution could be determined. The scenarios differ between: Starting in zero (1–4), at a lower level (5–8) and at a higher level (9–12); showing a faster increase (1, 3, 5, 7, 9, 11) or a slower increase (2, 4, 6, 8, 10, 12); and a higher (3, 4, 7, 8, 11, 12) or lower plateau (1, 2, 5, 6, 9, 10).

**FIGURE 5:**
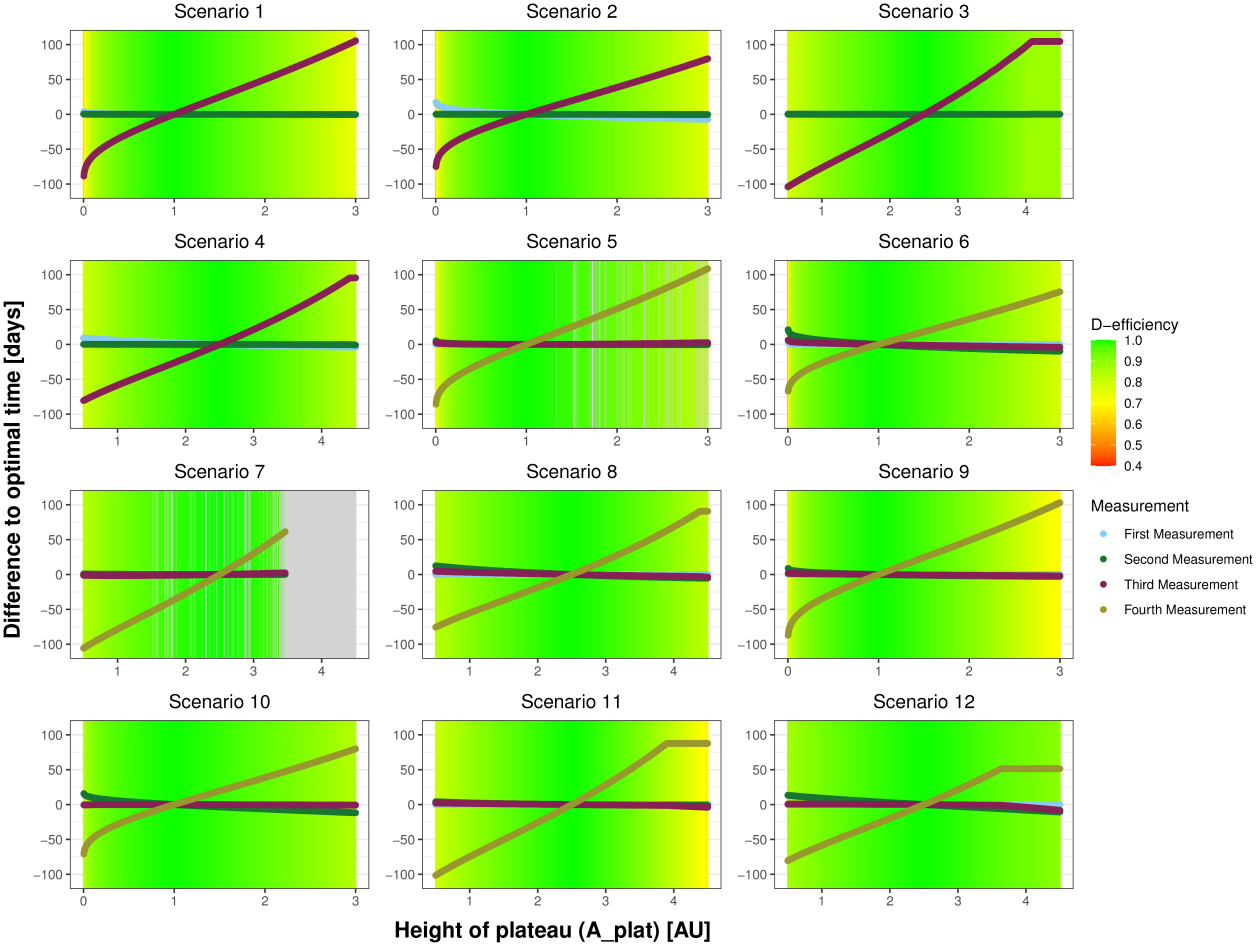
Plots illustrating the deviation of sampling times with respect to misspecification in the height of the plateau $A_{\text{plat}}$. Each plot represents one scenario. The lines represent the deviation of the optimal sampling times, based on misspecified initial information, compared to the optimal sampling times resulting by the “true” initial information. The color gradient highlights the D‐efficiency of the respective design, reflecting the effect of deviation of sampling times on the D‐efficiency. In Scenarios 1–4, three time points are optimized, while in Scenarios 5–12, four time points are optimized. The gray area indicates settings where no solution could be determined. The scenarios differ between: Starting in zero (1–4), at a lower level (5–8) and at a higher level (9–12); showing a faster increase (1, 3, 5, 7, 9, 11) or a slower increase (2, 4, 6, 8, 10, 12); and a higher (3, 4, 7, 8, 11, 12) or lower plateau (1, 2, 5, 6, 9, 10).

A closer examination, as shown in Figure 3, shows that misspecification in $A_{\text{plat}}$ has an effect on the D‐efficiency of the design across all scenarios with the minimal D‐efficiencies ranging from 0.55 to 0.86. However, misspecification in $t_{\max}$ mostly affects Scenario 9 (median 0.94, range: 0.47–1.0) and Scenario 11 (median 0.93, range: 0.43–1.0), which both involve a high starting value and a faster increase in antibody levels. Given that the time of the peak and the height of the plateau were identified as the most sensitive information, we further examined the impact of parameter misspecification on deviations from the optimal time‐points. Figure 4 illustrates the deviation and the D‐efficiency of the respective design for $t_{\max}$, while Figure 5 focuses on $A_{\text{plat}}$.

Misspecifications in $t_{\max}$ influence all measurements (see Figure 4) with the strongest effect observed for the second measurement for Scenarios 1–4 and the third measurement for Scenarios 5–12, respectively. The strongest impact on the D‐efficiency with misspecification in $t_{\max}$ is observed in Scenarios 9 and 11, as displayed in Figures 3 and 4. For all other scenarios, the impact of the misspecification of $t_{\max}$ on the D‐efficiency of the design is practically negligible. In contrast, we can observe that misspecification in $A_{\text{plat}}$ mostly influence a single measurement, namely the last measurement (see Figure 5), that is, the third measurement for Scenarios 1–4 and the fourth measurement for Scenarios 5–12. Depending on the magnitude of misspecification, deviation of up to 100 days can occur. If the height of the plateau is specified too low, the last measurement is scheduled too early and vice versa. However, we can see that in most scenarios, even this strong deviation only slightly worsens the D‐efficiency of the respective design. In Figure 3, Scenario 12 shows the most robust results, despite the fact that the deviation of the sampling times is similar to the other scenarios (see Figures 4 and 5). Further, most deviations from optimal sampling times show an approximately linear behavior, with a nonlinear behavior for more extreme values of $A_{\text{plat}}$.

There are numerical issues resulting from the misspecification in the initial information. The algorithm can either not determine the parameters of the resulting beta density or converge to optimal sampling times. Figure 4 shows numerical issues in Scenarios 3, 5, and 7, while Figure 5 shows numerical issues in Scenarios 5 and 7.

**FIGURE 6:**
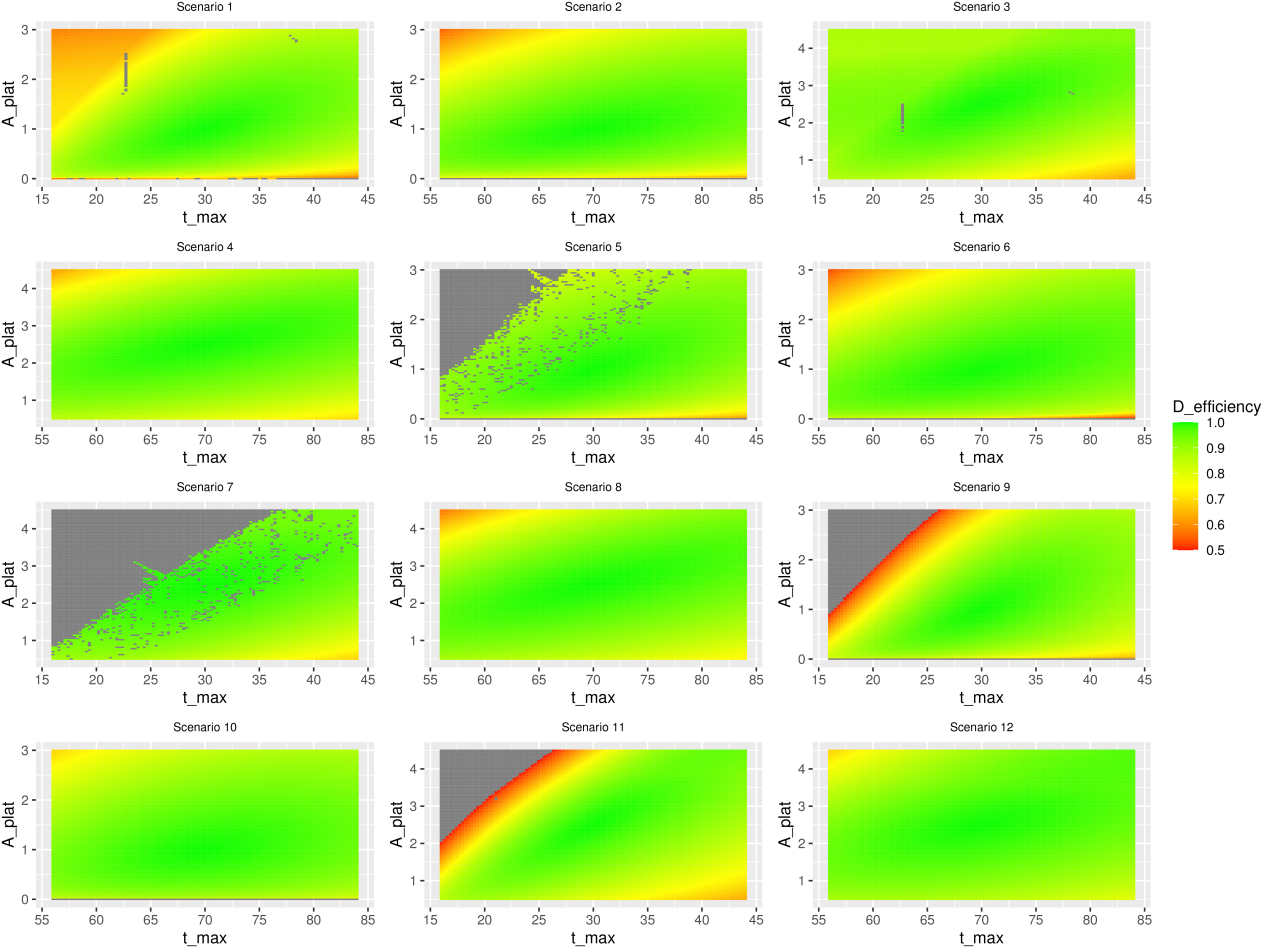
Graphical representation of misspecification in two parameters (the time of the maximum $t_{\max}$ and the height of the plateau $A_{\text{plat}}$) on the D‐efficiency of the respective design. The color gradient represents the D‐efficiency of the respective design, reflecting the effect of deviation of sampling times on the D‐efficiency. The gradient is caped at 0.5, while all values below 0.5 show the same color (red). In Scenarios 1–4, three time points are optimized, while in Scenarios 5–12 four times are optimized. The gray area indicates settings where no solution could be determined. The scenarios differ between: Starting in zero (1–4), at a lower level (5–8) and at a higher level (9–12); showing a faster increase (1, 3, 5, 7, 9, 11) or a slower increase (2, 4, 6, 8, 10, 12); and a higher (3, 4, 7, 8, 11, 12) or lower plateau (1, 2, 5, 6, 9, 10).

Table 3 and Figure 6 show the results for a double misspecification in the initial information, that is, in $t_{\max}$ and $A_{\text{plat}}$ simultaneously. Scenario‐wise the minimal D‐efficiency values range from 0.02 (Scenario 5) to 0.69 (Scenarios 7, 10, and 12), while the median D‐efficiencies range from 0.90 (Scenarios 1, 9, and 11) to 0.96 (Scenarios 7 and 12). Overall, the median D‐efficiency is 0.94. It is also observable, that numerical issues arise from a maximum that is misspecified as too early in combination with a plateau being specified as too high.

## 4 Discussion

Our novel framework facilitates the design of immunization studies by utilizing simple and intuitively understandable information. Assuming that antibody kinetics follow the functional form of the density of the beta distribution until reaching a plateau, the information of where to start, the timing and magnitude of the maximum concentration, and the timing and height of the plateau can be used to determine an optimal sampling schedule. The framework reduces the complexities of modeling antibody kinetics combined with the challenges of optimal design theory, by translating the needed initial guesses of parameters into easily interpretable input parameters, thereby lowering the barriers to practical implementation. The framework is feasible to design studies in which an initial increase is followed by a subsequent decay, an observed pattern in numerous immunization studies [33, 34, 35, 36, 37]. We have added an example of how the framework could be applied to Data S3 [38].

To analyze how the framework reacts to misspecification in this input parameters, we have conducted a robustness analysis, where we observed that when misspecifying one parameter at a time, the median D‐efficiencies remain above 0.95 and the first quartile is greater or equal to 0.9 for all parameters (see Table 3). This highlights the robustness of our proposed framework to misspecification in the initial information. Our analysis indicates that the height of the plateau and the time of maximum are the most sensitive to misspecification. Arguably, this can be attributed to the structure of the resulting curve. The height of the plateau is more important than its timing in the flat or stabilizing phase, whereas the timing of the maximum has a greater influence on the curve's steepness and overall structure than its height. Scenarios 9 and 11 warrant particular attention, as they show the highest degree of inefficiency under misspecification. Both scenarios involve higher starting levels and a faster increase, while differing in the height of the plateau. This suggests that the initial antibody concentration $A_{0}$ influences the design's D‐efficiency at least indirectly. Specifically, the effect on the D‐efficiency between zero ($A_{0}=0$) and lower values seems smaller than the difference between lower and higher values. Combining a higher initial antibody concentration with a faster increase, that is, an earlier maximum, potentially results in inefficient designs if the time of maximum is misspecified as too early. This result is intuitively reasonable, because if we start high and have a very early peak, this has the strongest effect on the structure of the curve. This indicates that subsequent analysis should examine the variables together. We further observed, that the deviation of the measurements are differently influenced by misspecification in different initial information. For misspecification in the height of the plateau (see Figure 5), mostly the last measurement is influenced, while for the time of the maximum, all measurements are influenced (see Figure 4). We again attribute this to the part of the curves where the respective information contributes the most. While changes in the time of the maximum influence the whole structure of the curve, changes in the height of the plateau mostly influence the later phase of the antibody kinetics. If we simultaneously misspecify the time of the maximum and the height of the plateau we see that the framework is also robust in terms of double misspecification. Depending on the respective scenario, some combinations of misspecification are less favorable than others. Especially the combination of a maximum misspecified as too early with a plateau misspecified as too high results in less efficient designs or even in non‐convergence. In some scenarios the choice of $A_{\text{plat}}=0$ results in general non‐convergence or almost zero D‐efficiency, which lets us conclude that it might be more reasonable to choose the lower limit of detection as minimally possible value.

Our proposed framework comes with some limitations. First, it relies on assumptions on the beta distribution, specifically that antibody kinetics exhibit an increase followed by a decrease, or in terms of parameters α>1 and β>1. If these assumptions are not met, we can not use the framework to design a study, because we can not use the property of the mode $x_{\max}$. Second, we assume known variability and correlation between measurements, and that all patients follow the same schedule. Assuming the same known variability for all measurements is a typical simplification at the stage of study design, while also a limitation and potential extension possibility of our framework. Other variance–covariance structures could be explored or include parameters to be estimated, however, this would lead to increased complexity in solving and would require additional initial information on those parameters. As the aim of this paper is to introduce the basic idea behind our framework, these assumptions, while simplifying the framework, leave room for future research. Further, we are working with mean values for the initial parameters, ignoring interindividual variability. This approach is, however, standard when working with locally optimal designs in study design. There is theory on the optimal design of nonlinear mixed effects models available, however, they present substantial practical challenges. For example, the FIM does not have a closed‐form solution in general, due to the fact that there is no analytical expression of the log‐likelihood function [39, 40, 41, 42]. We believe that, despite relevance for future research, we should first focus on other, more practically relevant issues, such as incorporating uncertainty in the initial information. In this paper we focus on a single schedule all patients should follow. Optimal designs consisting of multiple schedules, where patients are distributed across schedules in (predefined) ratios, may also further enhance design efficiency. However, since clinical studies often assign a single schedule to all patients, optimizing this single schedule would already improve the current status quo. Additionally, in some iterations we observe numerical issues when either determining the beta parameters or the optimal sampling times. We attribute this to mainly two reasons: either the beta density is not feasible to model the antibody kinetics (e.g., an almost flat curve or a very early maximum) or convergence issues using the implemented optimization algorithms. In the former case, we advise not to use the framework, while for the latter case we propose the pragmatic solution of sensitivity analysis, that is, varying the initial information slightly. We plan to automatically include sensitivity analysis in the implemented Shiny application in the upcoming version, since it should be used at the stage of study design anyway to assess numerical and analytical stability. Further research should also focus on stabilizing the numerical convergence by exploring different optimization algorithms and working on scaling and tolerances to address the issue of potentially small parameter values leading to non‐convergence. The convergence issues in Scenario 7, as seen in Figures 4 and 5, can be attributed to the fact that we observe non‐convergence when the shift term c reaches magnitudes of $10^{-8}$, which corresponds to the default numerical tolerances implemented in the “nleqslv” package.

Also the robustness analysis comes with limitations. Varying one parameter at a time is a simplification of the reality, as in practice, multiple parameters are likely misspecified simultaneously. Also, the values of our initial information chosen to represent different scenarios may not perfectly reflect practical conditions. We chose two values for $t_{\max}$ and $A_{\text{plat}}$ to distinguish between slower and faster increase and lower and higher plateaus. For $A_{0}$, we have chosen three values to not only distinguish between zero and nonzero starting values, but also different magnitudes of starting values. The chosen maximum antibody concentration is somehow arbitrary and can be interpreted as standardization, allowing to interpret the other antibody concentrations relative to the maximum. Nevertheless, the selected ranges align with typical observations, particularly on the log‐scale. We observed that the framework also works for different ranges, which in practice can be of a drastically higher magnitude (especially if working on the original scale). Further, clinical experience guided the chosen ranges of the misspecifications in the initial information, which might vary to a larger extent in practice. However, estimating the mean maximum antibody concentration within a range of ±20% is reasonable. Similarly, providing the timing of the maximum within ±2 weeks of the true value during study planning is a practical assumption, as is identifying whether patients have pre‐existing antibodies. Specifying the time of plateau (which is a simplification in itself) is reasonable in a time‐window of 100 days. For the ranges of misspecification in the initial antibody concentration and the height of the plateau, we have chosen the same deviation as for the height of the maximum and therefore relatively wider ranges of misspecification. Additionally, clinical trial simulations should be conducted to evaluate the benefit of an optimal design compared to a misspecified optimal design compared to a non optimized design in terms of bias and variability in parameter estimates.

The main goal of this framework is to provide a tool for clinicians to design an optimal sampling schedule for studies focusing on antibody kinetics. We are therefore currently developing an R‐Shiny application, allowing the user to easily apply the framework [43]. Furthermore, we are working on incorporating double exposure scenarios, which are commonly seen in practice in studies where the immunization consists of primary and secondary vaccine doses. Additionally, we want to allow for uncertainty in the initial information, to account for statements like: the maximum is somewhere between Weeks 3 and 5. Several potential approaches can address this issue. A practical and pragmatic method is to conduct sensitivity analysis across the range of parameter uncertainty, evaluating how the resulting optimal designs vary. A more analytical approach would be to explicitly incorporate prior uncertainty of the parameters into the optimality criterion, for example, by ED‐optimal designs or other robust design methods [44, 45]. Often clinical studies aim to determine whether the antibody kinetics differ between groups; extending the framework to allow for covariates would therefore be of practical interest.

With the utmost caution, based on the results of the robustness analysis, we propose the following practical recommendations: To determine to what extent the study population has pre‐existing antibodies, we recommend collecting a sample, if possible, prior to the immunization event (corresponds to Time 0 in Table 2). Additionally, collecting a second early sample appears to enhance the efficiency of the study design. In most scenarios, a sample is recommended to be taken near the expected maximum and, if misspecified, near the corresponding misspecified value (see Table 2 and Figure 4).

In conclusion, the proposed framework demonstrates robustness with respect to misspecifications in the initial information, particularly when misspecifications are marginal. Despite its limitations, this framework provides a good starting point for applying optimal design theory in immunization studies, where describing antibody kinetics is the main objective. Its major advantage is that it uses interpretable information, making it accessible for healthcare professionals. This represents an important step forward in optimizing sampling schedules for blood samples in immunization studies, in an area where little attention has been given to date.

## Conflicts of Interest

The authors declare no conflicts of interest.

## Supporting information


**Data S1:** sim70293‐sup‐0001‐Supinfo1.R.

**Data S2:** sim70293‐sup‐0002‐Supinfo2.R.

**Data S3:** sim70293‐sup‐0003‐Supinfo3.pdf.

## Data Availability

Data sharing not applicable to this article as no datasets were generated or analysed during the current study.
