# Supplementary material for: A Practical Framework to Design Immunization Studies Based on the Beta Distribution
Source: Stat Med. 2025 Oct 7;44(23-24):e70293. doi: 10.1002/sim.70293 (PMC12503089; doi:10.1002/sim.70293)
Supplement: Supplementary file 3 — Data S3: sim70293‐sup‐0003‐Supinfo3.pdf. [file SIM-44-0-s001.pdf]

Received: Added at production | Revised: Added at production | Accepted: Added at production

DOI: xxx/xxxx

SUPPLEMENTARY MATERIAL

Supplementary Material: A practical framework to design immunization studies based on the beta distribution

Stefan Embacher[1] | **Andrea Berghold**[1] | **Kirsten Maertens**[2] | **Sereina A. Herzog**[1]

[1]Institute for Medical Informatics, Statistics and Documentation, Medical University of Graz, Graz, Austria

[2]Centre for the Evaluation of Vaccination, Vaccine and Infectious Diseases Institute, University of Antwerp, Antwerp, Belgium

Correspondence
stefan.embacher@medunigraz.at

Present address
Auenbruggerplatz 2, 8036 Graz, Austria

S1 - PARTIAL DERIVATIVES OF SIMPLIFIED FRAMEWORK

The partial derivatives of the beta density, in the case we start with antibody concentration of zero ($A(0) = 0$) are given by

$$\begin{cases} \dfrac{\partial \mu}{\partial \alpha} = \dfrac{1}{B(\alpha,\beta)} \Bigg[log\left(\dfrac{t}{t_{scale}}\right)\left(1-\dfrac{t}{t_{scale}}\right)^{\beta-1}\left(\dfrac{t}{t_{scale}}\right)^{\alpha-1} \\ \qquad -(\psi(\alpha)-\psi(\alpha+\beta))\left(1-\dfrac{t}{t_{scale}}\right)^{\beta-1}\left(\dfrac{t}{t_{scale}}\right)^{\alpha-1}\Bigg] \\ \dfrac{\partial \mu}{\partial \beta} = \dfrac{1}{B(\alpha,\beta)} \Bigg[log\left(1-\dfrac{t}{t_{scale}}\right)\left(1-\dfrac{t}{t_{scale}}\right)^{\beta-1}\left(\dfrac{t}{t_{scale}}\right)^{\alpha-1} \\ \qquad -(\psi(\beta)-\psi(\alpha+\beta))\left(1-\dfrac{t}{t_{scale}}\right)^{\beta-1}\left(\dfrac{t}{t_{scale}}\right)^{\alpha-1}\Bigg] \\ \dfrac{\partial \mu}{\partial t_{scale}} = \dfrac{1}{B(\alpha,\beta)t_{scale}^2} \Bigg[(\beta-1)t\left(1-\dfrac{t}{t_{scale}}\right)^{\beta-2}\left(\dfrac{t}{t_{scale}}\right)^{\alpha-1} \\ \qquad -(\alpha-1)t\left(1-\dfrac{t}{t_{scale}}\right)^{\beta-1}\left(\dfrac{t}{t_{scale}}\right)^{\alpha-2}\Bigg] \end{cases}$$

Alternative corresponding author: Sereina A. Herzog (sereina.herzog@medunigraz.at)

© Copyright Holder Name

S2 - ANALYTICAL AND NUMERICAL SENSITIVITY OF σ AND ρ

To show that the choice of σ and ρ do not affect the optimal sampling times, except for numerical tolerance, we need the assumption that $n = p$, such that the Jacobian matrix of μ with respect to θ is a square matrix of full rank, which we fixed throughout the robustness analysis. In our setting, we assumed that our covariance matrix is independent of θ, specifically that it is of the form $\Sigma = S * AR(1)$, where $S = diag(\sigma^2)$ and $AR(1)_{ij} = \rho^{|i-j|}$ for $i, j = 1, \ldots, n$, with $0 < \rho < 1$ and $n > 1$. The following result, however, holds for a general covariance matrix Σ which is independent of θ. Because we work with multivariate normally distributed observations, it is valid to assume that Σ is a regular, positive definite matrix of dimension $n \times n$. The FIM can be written in matrix form in the following way:

$$FIM = H^T \Sigma^{-1} H,$$

where $H = D_\theta \boldsymbol{\mu}(\boldsymbol{t}, \boldsymbol{\theta})$, which is a square matrix of dimension $n \times n$. Therefore it follows, because H and Σ are square matrices of the same order, that

$$\det(FIM) = det(H^T \Sigma^{-1} H) = \frac{1}{det(\Sigma)} \det(H^T H)$$

which results in the optimization of the following expression with respect to $\boldsymbol{t} = (t_1, \ldots, t_n)$

$$-\ln\left(\frac{1}{det(\Sigma)} \det(D_\theta \boldsymbol{\mu}(\boldsymbol{t}, \boldsymbol{\theta})^T D_\theta \boldsymbol{\mu}(\boldsymbol{t}, \boldsymbol{\theta})) + 1\right).$$

Therefore the determinant can be considered a constant, non-zero scaling factor in the convex optimization problem and hence does (analytical) not affect the optimal solution. To assess the numerical sensitivity, we have conducted a small robustness analysis regarding the choice of σ and ρ. We set $\sigma \in \{0.2, 0.4, 0.6, 0.8, 1, 1.2, 1.4, 1.6, 1.8, 2\}$ and $\rho \in \{0, 0.2, 0.4, 0.6, 0.8\}$ resulting in a total of 50 possible combinations. For each scenario and each pair, we have optimized the sampling times and calculated the difference between the maximal and minimal value (for each scenario) in order to quantify the numerical variability resulting by the choice σ and ρ. The corresponding observed differences are given in Table 1, where we observe the overall largest difference to be 0.0256635 days, which corresponds to 36 minutes. Therefore, we conclude that the numerical variability can be ignored in practice.

TABLE 1 The maximal difference [days] of optimal sampling times in 12 scenarios, across 50 different pairs of standard deviation and correlation.

Scenario	Time 1 difference	Time 2 difference	Time 3 difference	Time 4 difference
1	3.03e-05	0.0009585	0.0041970	NA
2	9.15e-05	0.0006000	0.0018000	NA
3	6.30e-06	0.0022200	0.0138315	NA
4	1.80e-04	0.0016500	0.0031652	NA
5	0.00e+00	0.0000750	0.0014534	0.0085500
6	0.00e+00	0.0004500	0.0016650	0.0030000
7	0.00e+00	0.0004500	0.0016650	0.0030000
8	0.00e+00	0.0004230	0.0026717	0.0105150
9	0.00e+00	0.0001801	0.0018000	0.0076515
10	0.00e+00	0.0045000	0.0165298	0.0150000
11	0.00e+00	0.0000600	0.0029835	0.0256635
12	0.00e+00	0.0021000	0.0030000	0.0150000

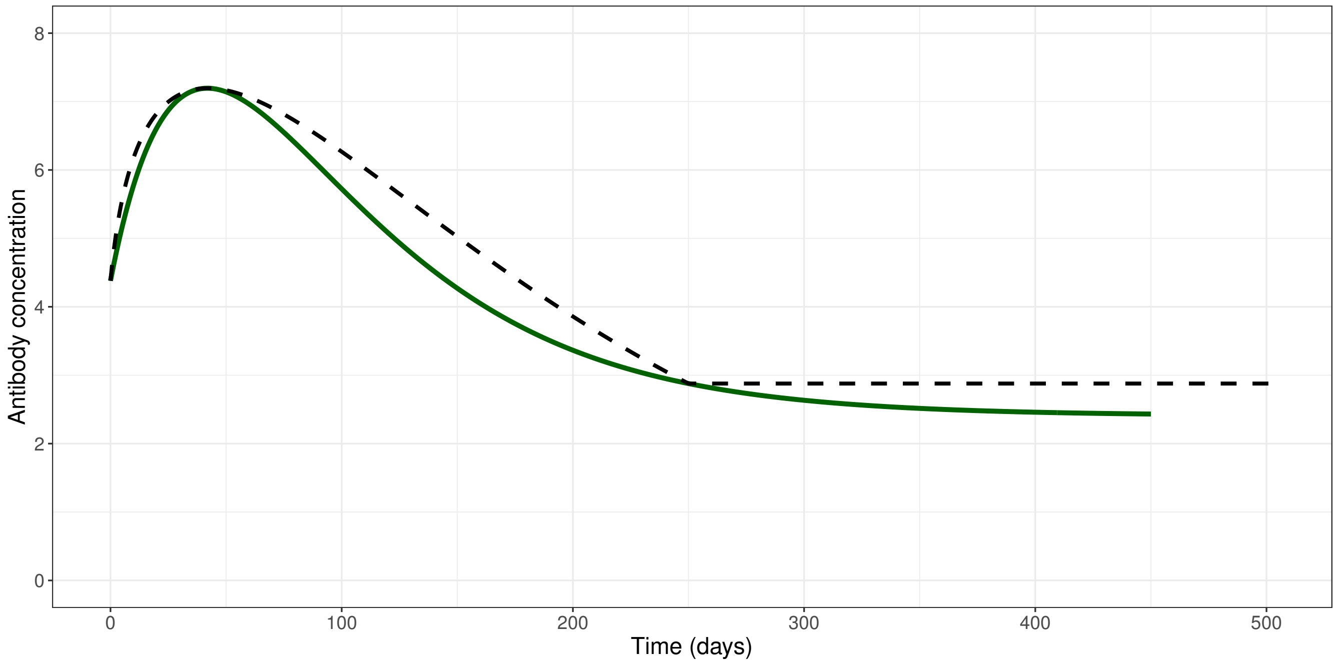

FIGURE 1 The original model as published by Violan et al.,[1] is shown as the darkgreen line. The black dashed line represents the curve resulting, under the assumptions made, from our framework.

S3 - EXAMPLE

To provide an example of how the framework can be used, we plan a study with the main objective to model the antibody kinetics after SARS-Cov-2 infection. The initial information for our study design is based on the results reported by Violán et al.,[1] and we want to investigate IgG against the nucleocapsid protein (IgG(N)). The modelling results by Violán et al. are provided in their supplementary material allowing us to obtain the numerical values for A_0, A_{max} and t_{max} directly.[1] We use the supplied general equation because we cannot determine if we are dealing with asymptomatic, mild, or severe disease at the time of designing the study. The equation is given as

$$IgG(N)(t) = 2.41 + 1.97\exp(-10.85t) - 26.15(\exp(-10.85t) - \exp(-7.03t)),$$

where t is normalized for the maximum length of follow-up, which is 450 days for IgG(N). Based on the equation, the following input values for the initial information are obtained: $A_0 = 4.38$, $t_{max} = 42$ and $A_{max} = 7.195$. Upon graphical inspection of the curve displayed in Figure 1, we assumed to reach the plateau at day 250, i.e., $t_{plat} = 250$ corresponding to $A_{plat} = 2.878$. Under the assumption of a standard deviation of 1 and a moderate correlation of 0.5, samples would be taken on day 0, day 8.88, day 70.71, and day 250, as shown in Figure 2. In our hypothetical study, we impose specific time constraints on the time-windows for sample collection to ensure well monitoring of patients with infections. Participants are required to attend regular hospital visits, with the first sample collected within the first week post-infection, the second within the second week, and the third approximately one month after infection. To end the study within six months of infection, the fourth sampling time-window is restricted accordingly. Given these restrictions, we get the following output from the Shiny App: *"When assuming that the antibodies at time 0 are 4.38, the maximum of 7.195 is reached at day 42 and a plateau at 2.878 is reached at day 250, the optimal sampling times in (continuous) days are given as: Day 0, Day 8, Day 38, Day 180. We restricted the sampling times to be within days [0, 7], [8, 14], [25, 38], [39, 180], respectively, while assuming that the structure of the variance-covariance matrix is that of an AR(1)-process with standard deviation 1 and a rho of 0.5."*

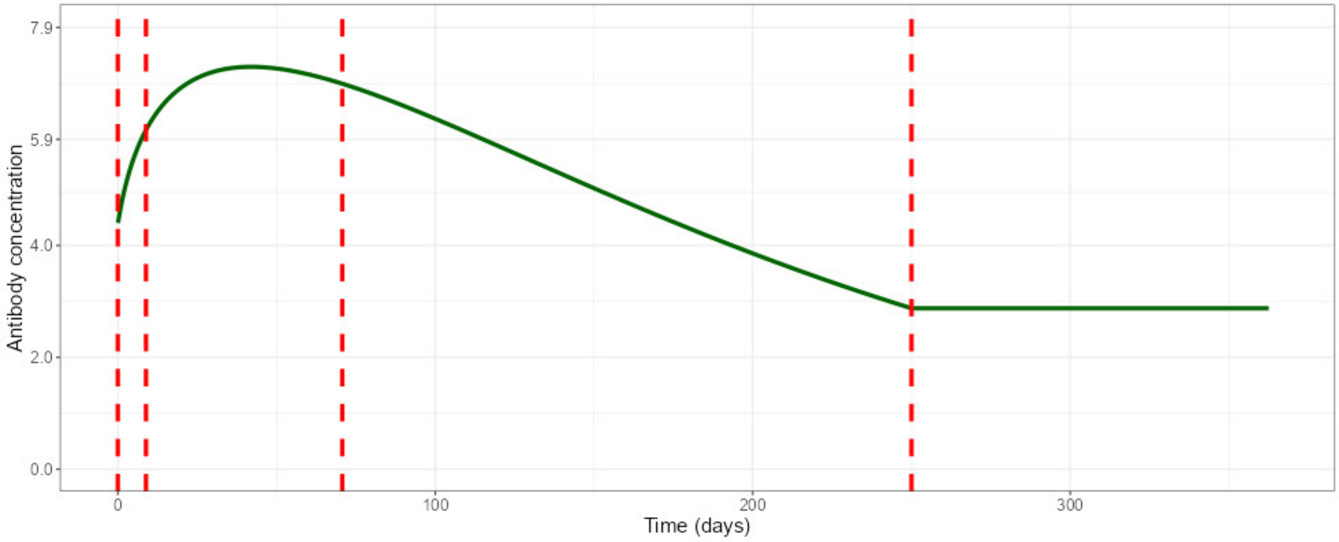

FIGURE 2 The green curve represents the curve resulting by our framework, while the red dashed lines indicate the optimal sampling times without restrictions on the time-windows as described in the example.

S4 - SENSITIVITY ANALYSIS OF STARTING VALUES

In order to assess whether parameter uncertainty exists in the solutions of the beta distribution, we sampled the starting values from the uniform distribution, given the following limits:

$$\alpha_{start} \sim U(1.01, 100)$$
$$\beta_{start} \sim U(1.01, 100)$$
$$t_{scale\_start} \sim U(t_{plat}, 50000)$$
$$c \sim U(0.01, 100)$$

where c is only used if $A_0 \neq 0$. We ran 10000 simulation for each of the 12 scenarios. We examine the numerical variability by calculating the difference between the maximal and the minimal value for each of the parameters for each scenario. The largest observed difference was of the magnitude 10^{-5}, indicating that the choice of the starting value has minimal impact on the numerical solutions for α, β, t_{scale} and c. We therefore conclude that there is no evidence of local optima in our optimization problem. However, the choice of the starting values did affect the convergence behaviour.

S5 - PLOT SCENARIOS WITH OPTIMAL TIMES

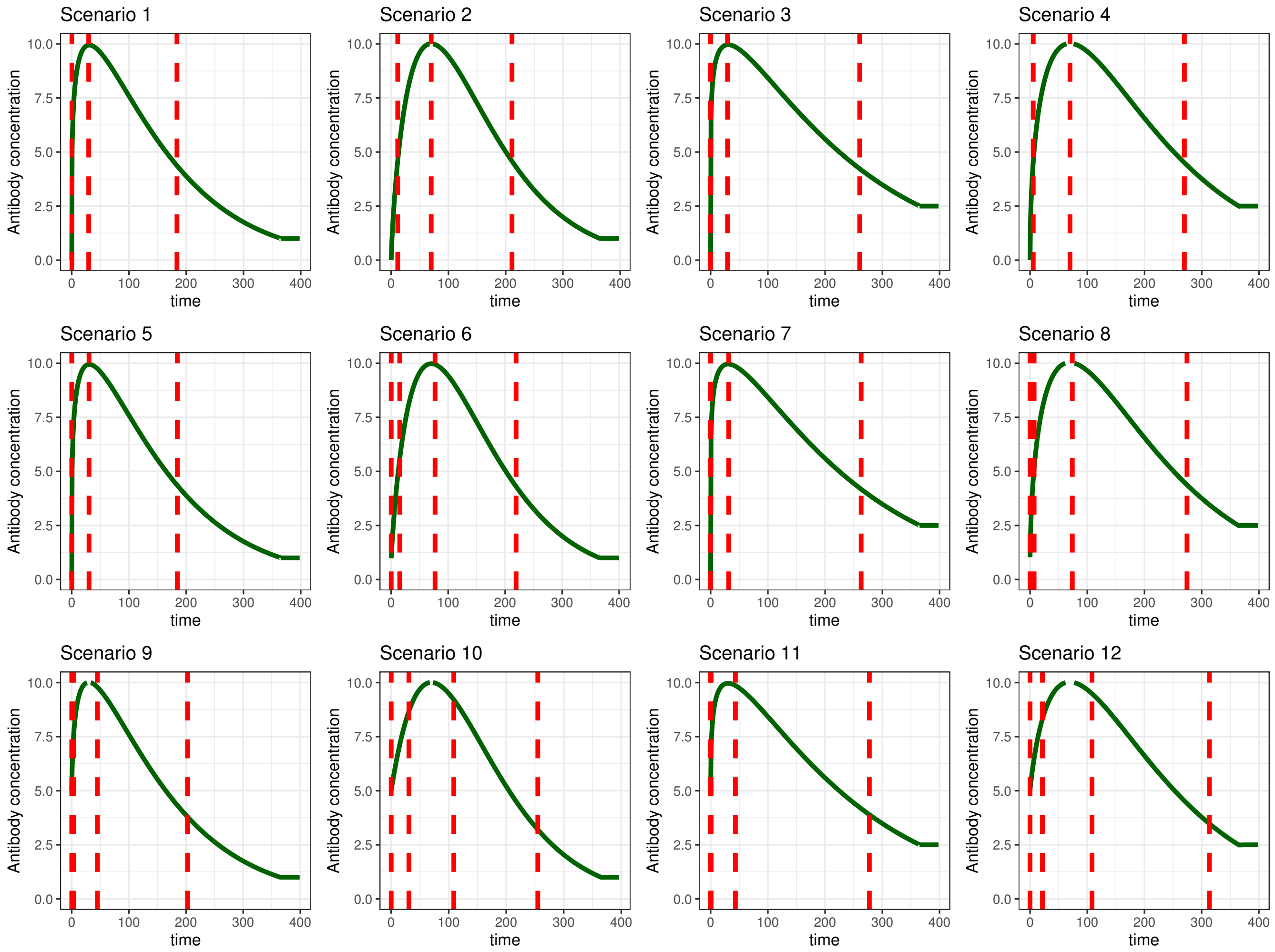

FIGURE 3 The green curve shows the antibody concentration in the 12 different scenarios, while the dashed red lines indicate the optimal sampling times. The scenarios differ between: starting in zero (1-4), at a lower level (5-8) and at a higher level (9-12); showing a faster increase (1,3,5,7,9,11) or a slower increase (2,4,6,8,10,12); and a higher (3,4,7,8,11,12) or lower plateau (1,2,5,6,9,10).

REFERENCES

1. Violán C, Torán-Monserrat P, Quirant B, et al. Kinetics of humoral immune response over 17 months of COVID-19 pandemic in a large cohort of healthcare workers in Spain: the ProHEpiC-19 study. *BMC Infectious Diseases.* 2022;22(1):721. doi: 10.1186/s12879-022-07696-6
